# Relationship between genome‐wide and MHC class I and II genetic diversity and complementarity in a nonhuman primate

**DOI:** 10.1002/ece3.9346

**Published:** 2022-10-13

**Authors:** Rachel M. Petersen, Christina M. Bergey, Christian Roos, James P. Higham

**Affiliations:** ^1^ Department of Anthropology New York University New York New York USA; ^2^ New York Consortium in Evolutionary Primatology New York New York USA; ^3^ Department of Genetics and the Human Genetics Institute of New Jersey Rutgers University Piscataway New Jersey USA; ^4^ Gene Bank of Primates and Primate Genetics Laboratory German Primate Center Leibniz Institute for Primate Research Göttingen Germany

**Keywords:** complementarity, ddRAD sequencing, heterozygosity, mate choice, MHC

## Abstract

Although mate choice is expected to favor partners with advantageous genetic properties, the relative importance of genome‐wide characteristics, such as overall heterozygosity or kinship, versus specific loci, is unknown. To disentangle genome‐wide and locus‐specific targets of mate choice, we must first understand congruence in global and local variation within the same individual. This study compares genetic diversity, both absolute and relative to other individuals (i.e., complementarity), assessed across the genome to that found at the major histocompatibility complex (MHC), a hyper‐variable gene family integral to immune system function and implicated in mate choice across species. Using DNA from 22 captive olive baboons (*Papio anubis*), we conducted double digest restriction site‐associated DNA sequencing to estimate genome‐wide heterozygosity and kinship, and sequenced two class I and two class II MHC loci. We found that genome‐wide diversity was not associated with MHC diversity, and that diversity at class I MHC loci was not correlated with diversity at class II loci. Additionally, kinship was a significant predictor of the number of MHC alleles shared between dyads at class II loci. Our results provide further evidence of the strong selective pressures maintaining genetic diversity at the MHC in comparison to other randomly selected sites throughout the genome. Furthermore, our results indicate that class II MHC disassortative mate choice may mediate inbreeding avoidance in this population. Our study suggests that mate choice favoring genome‐wide genetic diversity is not always synonymous with mate choice favoring MHC diversity, and highlights the importance of controlling for kinship when investigating MHC‐associated mate choice.

## Graphical abstract

This study aims to understand congruence in genome‐wide and MHC‐specific genetic variation within the same individual. We found that genome‐wide diversity was not associated with MHC diversity at either MHC class type. Additionally, kinship was a significant predictor of the number of MHC alleles shared between dyads at class II MHC loci.

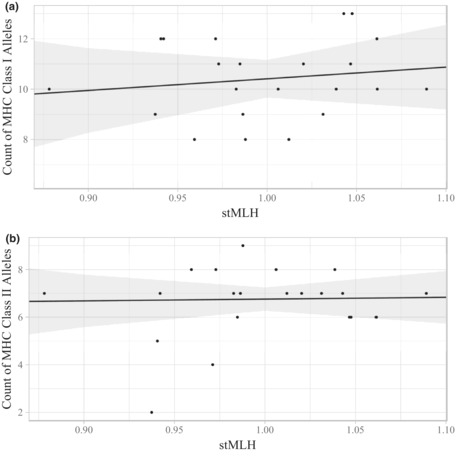

## 1 INTRODUCTION

Mate choice favoring both absolute genetic variation and variation relative to the mating individual (i.e., complementarity) may provide indirect benefits to offspring in the form of genetic diversity and its associated fitness advantages (Kempenaers, 2007). This type of diversity‐based mate choice may occur at the genome‐wide scale, favoring traits such as high global heterozygosity or low genetic relatedness (Hoffman et al., 2007; Ilmonen et al., 2009), or at the scale of particular functional loci that may impact fitness (Landry et al., 2001; Neff et al., 2008; Winternitz et al., 2017). Differences in the selective pressures maintaining genetic diversity at these two levels may complicate mate choice investigations, as genetic diversity measured at the genome‐wide level may differ from the diversity present at a specific functional site.

One such functional site is the major histocompatibility complex (MHC), a highly polymorphic gene family thought to be under pathogen‐mediated balancing selection and influential in processes of mate choice due to its role in immune system function (Piertney & Oliver, 2006). The maintenance of high genetic diversity at the MHC region is often attributed to a heterozygote advantage, whereby different MHC alleles possess different antigen‐binding capacities (Hughes & Yeager, 1998), and more diverse genotypes provide protection against a broader array of immune challenges (Doherty & Zinkernagel, 1975; Potts & Slev, 1995). Simultaneously, extreme diversity at the MHC may also impose a survival disadvantage, as self‐reactive T‐cells must be destroyed to avoid autoimmune disease, restricting T‐cell repertoire in individuals with highly diverse MHC regions (Kubinak et al., 2012). This process may ultimately constrain runaway selection on MHC diversity, and instead favor individuals with intermediate levels of diversity or locally adapted gene complexes, a process which has been experimentally confirmed in some taxa (Eizaguirre et al., 2012; Wegner et al., 2003).

Although an association between genome‐wide and MHC diversity has been observed in small, inbred, or bottlenecked populations (Miller & Lambert, 2004), it is generally thought that measures of genome‐wide diversity will not be correlated with MHC diversity, as the evolutionary mechanisms thought to maintain the former (genetic drift) differ from mechanisms thought to maintain the latter (balancing selection). As a consequence, mate choice favoring partners with high genome‐wide diversity may or may not reinforce mate choice favoring genetic diversity at the MHC. Some mate choice studies have attempted to address this potentially confounding interaction between genome‐wide and MHC diversity by investigating how MHC heterozygosity and complementary relate to genome‐wide heterozygosity and kinship estimated using a small number of microsatellites (5 loci: Landry et al., 2001; 7 loci: Schwensow et al., 2008; 16 loci: Huchard et al., 2010; 20 loci: Zhang et al., 2020). Although microsatellites perform well in a variety of analytical contexts, genotyping at a small number of microsatellites can be an unreliable proxy of overall genomic heterozygosity (Väli et al., 2008), which may be influenced by population history and degree of microsatellite polymorphism (Miller et al., 2014). Next generation sequencing technologies, such as double digest restriction site‐associated DNA (ddRAD) sequencing, allow genotyping of tens of thousands of single nucleotide polymorphisms (SNPs). Coupled with direct sequencing of known functional loci, high‐density SNP sampling allows an avenue by which to investigate the roles of functional loci versus genome‐wide genetic attributes in the process of mate choice.

The social and mating system of the olive baboon (*Papio anubis*) make it an excellent species to evaluate processes of genetically based mate choice. Olive baboons live in large multimale, multifemale groups, and display polygynandrous mating and male‐biased dispersal, a pattern that is fairly common among primates (Pusey & Packer, 1986; Smith, 1992). Females exhibit both direct and indirect mate choice by performing sexual solicitations to particular males (Walz, 2016), and by actively inciting copulations through copulation calls and conspicuous sexual swellings (Higham et al., 2008; Maestripieri & Roney, 2005). Sperm competition is also evident from males' large relative testes volume (Jolly & Phillips‐Conroy, 2003). This interesting combination of traits suggests a possible role for pre‐ and post‐copulatory MHC‐associated mate choice in both males and females. As a first step towards a better understanding of genetically based mate choice in the olive baboon, we here characterize the relationship between genome‐wide versus MHC‐diversity and complementarity in this species. Although a basic understanding of MHC diversity in the genus *Papio* is beginning to accumulate (Huchard et al., 2006; Morgan et al., 2018; van der Wiel et al., 2018), it remains unclear how MHC genotype relates to genome‐wide diversity and complementarity, which is a potentially confounding factor when trying to identify genetic targets of mate choice.

To expand our current understanding of how genome‐wide measures of heterozygosity and complementarity relate to those of the MHC region, we characterize thousands of polymorphic sites throughout the genomes of a population of olive baboons, and sequence four highly variable MHC loci representing two different classes of MHC receptors. By sequencing genes of different MHC classes, this study also has the opportunity to test for associations between class I and class II MHC diversity, a potential mechanism by which optimal genetic diversity at the MHC may be maintained. Our study has five aims. We aim to: (1) **characterize global heterozygosity and kinship** within study subjects; (2) **characterize MHC heterozygosity and complementarity** within and between study subjects; (3) assess potential **associations between diversity at different MHC classes**; (4) determine the **relationship between global and MHC heterozygosity**; and (5) determine the **relationship between kinship and MHC complementarity**. Together, our study represents an attempt to better understand the relationship between genome‐wide and MHC diversity and complementarity, both of which may be relevant to the study of indirect benefits mate choice.

## 2 METHODS

### 2.1 Ethical note

This study was reviewed and approved by the Ministry of National Education, Higher Education, and Research in France, and New York University's IACUC committee (MNEHER agreement C130877 and NYU protocol # 18‐1504).

### 2.2 Dataset generation

#### 2.2.1 Sampling and DNA extraction

We obtained whole blood samples from 22 olive baboons (7 males, 15 females) living at Le Centre National de la Recherche Scientifique Station de Primatologie (CNRS SdP) in Rousset‐sur‐Arc, France. The pedigree information that is known for these individuals is available in Table S1. Seventeen of the 22 study individuals originated from a population once housed at the St. Vrain Zoo in Essone, France, where baboons were housed in large multimale multifemale social groups. We used the function “pwr.f2.test” in the R package “pwr” v1.3 (Champely, 2020) to determine the model parameter values necessary to obtain different target power with our sample size. We inputted the numerator degrees of freedom (number of predictor variables), denominator degrees of freedom (number of observations minus the number of predictor variables plus 1), significance level cutoff (*p* = .05), and three different power values (.8, .5, and .1) to determine the effect size detectible at each target power. We confirmed that our study's sample size has strong statistical power (≥80%) to detect large effect sizes (*f* ^2^ ≥ 0.39), moderate to large power (≥50%) to detect moderate effect sizes (*f* ^2^ ≥ 0.19), and weaker power (≥10%) to identify small effect sizes (*f* ^2^ ≥ 0.02). Thus, small effect sizes may not be reliably detected with our current sample size.

We extracted DNA using either the Qiagen QIAamp DNA mini kit (*N* = 7; 4 females and 3 males) or GEN‐IAL First‐DNA All tissue kit (*N* = 15; 11 females and 4 males) following manufacturer's instructions. We quantified DNA using a Qubit 3.0 fluorometer and assessed DNA purity using a Nanodrop ND‐1000.

#### 2.2.2 Genome‐wide SNP genotyping

We prepared ddRAD sequencing libraries following Peterson et al. (2012). We digested 1 μg of DNA using 10 units of two restriction enzymes (SphI and MluCI), and excluded fragments outside of a 185 ± 19 bp target window using the automated Blue Pippin System and 2% Agarose Gel Cassettes. We ligated Illumina platform adapters customized for enzyme cut sites and cleaned products using AMPure XP beads. We individually indexed samples using NEBNext Multiplex Oligos for Illumina sequencing, conducted a second round of size selection, and sequenced the libraries on one lane of the Illumina HiSeq 2500 with 150 bp paired end reads (comprehensive methods available in the Supplementary Materials).

#### 2.2.3 MHC sequencing

We amplified and sequenced functionally significant regions of four MHC receptor types, representing two classes of MHC molecules: MHC A and B (class I), and MHC DQ and DR (class II). Within class I loci, we sequenced a 195 bp segment within the α_1_ domain involved in antigen binding, and within class II loci, we sequenced a 188 bp segment within the α_1_ domain of DQ receptors (i.e., DQA) and a 252 bp segment within the β_1_ domain of DR receptors (i.e., DRB). These segments comprise portions of the antigen‐binding cleft, and hence include amino acids that are functionally important for the recognition and binding of intra and extracellular pathogens.

To amplify the desired sequences, we used the MilliporeSigma FastStart High Fidelity PCR System, 50 ng of template DNA, and primers (Metabion) described in Table S1. Following PCR amplification, we conducted gel electrophoresis and excised bands at the appropriate length for each amplicon. We estimated the concentration of the PCR products using a Qubit 3.0 fluorometer, estimated molarity, and performed an indexing PCR using Hot Start Pfu DNA Polymerase. We then purified the indexed products using AMPure XP beads, and pooled samples into a final 3 nM library for sequencing on Illumina's MiSeq with v2 chemistry and 200 bp paired end reads (comprehensive methods available in the Supplementary Materials).

### 2.3 Bioinformatic analyses

#### 2.3.1 Genome‐wide SNP genotyping

We cleaned and filtered ddRAD sequences using the program STACKS v.2 (Rochette et al., 2019), excluding reads with low‐quality scores or without both enzyme cut sites. We mapped the filtered reads to the *Papio anubis* reference genome (NCBI Panu v. 3.0; accession no GCA_000264685.2) using the Burrows‐Wheeler Aligner “mem” algorithm with default parameters (Li, 2013). We performed shared SNP calling using the STACKS reference mapping pipeline (Rochette et al., 2019), requiring loci to be present in at least 80% of individuals to be included in the SNP catalog. To generate a more accurate estimate of genome‐wide heterozygosity for each individual, we excluded SNPs in strong linkage disequilibrium (LD) by scanning sequences in a sliding window and removing at random SNPs with a probability of LD (*r* ^2^) ≥ .5 (‐‐indep‐pairwise 50 5 0.5 in PLINK; Purcell et al., 2007). These sequence reads have been submitted to NCBI's Sequence Read Archive (SRA) and are available under project number PRJNA875430.

To assess whether our study population displays the genetic signatures of a bottleneck or inbreeding, both of which may influence genetic diversity (Groombridge et al., 2000; Wisely et al., 2002), we compared the genetic diversity present in our study population to that observed in wild populations by merging our SNP dataset with two previously published *Papio* datasets (Bergey, 2015; Rogers et al., 2019). Once again, we pruned sites in linkage disequilibrium and estimated individual heterozygosity in PLINK (Purcell et al., 2007).

#### 2.3.2 MHC sequencing

We filtered MHC sequences for quality using Trimmomatic (Settings: Leading:28, Trailing:28, Window:4:25, MinLen: 150; Bolger et al., 2014), and merged forward and reverse reads using PandaSeq v.2.11 (Masella et al., 2012). We mapped reads to known olive baboon MHC‐A, B, DQA, and DRB loci taken from the IPD‐MHC database (www.ebi.ac.uk/ipd/mhc), split by contig, and filtered for target length of each amplicon. To identify PCR and sequencing artifacts, we performed rare K‐mer filtering, using a scanning window of 15 bp, and excluded sequences containing 12 K‐mers in a row falling beneath 15% of the median read coverage. We then collapsed identical sequences and kept only sequences comprising at minimum 5% of the total number of filtered reads for that locus (Barbian et al., 2018). Additionally, we kept sequences that had a copy number >1000 that were also present at >5% of total copy number in another individual. We chose this threshold because most loci displayed a natural 10 to 100‐fold drop in sequence copy number when comparing the last sequence present at >1000 copies versus the first sequence present at <1000 copies (Figure S1). Within MHC‐DRB, the most thoroughly characterized loci of the four studied here, 26 out of the 27 retained sequences had previously been identified in other studies, while those falling below the 5% copy number threshold had not been previously identified, suggesting that reads falling below our filtering criteria were likely generated from sequencing error.

After filtering, we aligned the retained reads to all known *Papio anubis* MHC sequences using BLAST (Altschul et al., 1990), and identified previously known sequences as those matching published sequences with a 100% identity and 0 gaps. Sequences possessing <100% identity or >0 gaps were distinguished as new MHC alleles. These sequence data have been submitted to GenBank (accession numbers OP375715–OP375798).

#### 2.3.3 MHC supertype analyses

We defined MHC supertypes using the physiochemical properties of amino acids located at positively selected sites (PSS). We identified PSS by comparing rates of synonymous (dS) to non‐synonymous (dN) nucleotide substitutions in protein coding regions using methods described by Goodswen et al. (2018). The dN/dS method was originally proposed to identify selective pressures in distantly diverged sequences of independent lineages, and is less sensitive to identifying selective pressures in sequences derived from individuals within the same population (Kimura, 1979; Kryazhimskiy & Plotkin, 2008). However, within the MHC gene family, amino acid polymorphisms are most often found at locations involved in antigen binding, and thus this measure of positive selection provides insight into the location of functionally important amino acid sites (Bjorkman et al., 1987; Brown et al., 1988).

We determined open reading frames by performing a BLAST alignment to the IPD‐MHC database. We translated sequences in R using the package “seqinr” v4.2.8 (Charif & Lobry, 2007) and performed multiple protein sequence alignment in MAFFT v7 (Katoh & Standley, 2013). We converted protein alignments into codon alignments using PAL2NAL v14 (Suyama et al., 2006), and constructed a maximum likelihood tree of the alignments using RAxML v2 (Stamatakis, 2014) and a generalized time reversible (GTR) GAMMA substitution model, with the best‐scoring tree selected using 100 bootstrap iterations. We then computed substitution rate ratios (dN/dS) by inputting the PAL2NAL codon alignment and RAxML tree into the CODEML program of the PAML v4.9 package (Yang, 2007). This software identifies statistically significant PSS using the Bayes Empirical Bayes (BEB) analysis computed under NSsite model 8 (Yang et al., 2005).

Next, we aligned the amino acids associated with each PSS and described the physiochemical properties of each site in the form of five *z*‐descriptors: *z*_1_ (hydrophobicity), *z*_2_ (steric bulk), *z*_3_ (polarity), *z*_4_ and *z*_5_ (electronic effects; Sandberg et al., 1998). We compiled a mathematical matrix containing the five *z*‐scores of each PSS of each allele and performed an agglomerative hierarchical clustering analysis using Euclidian distance and the average linkage method with the R function “hclust” in the “stats” package v4.1.2 (R Core Team, 2021). We used the R package “dynamicTreeCut” v1.63.1 (Langfelder et al., 2014) to identify significant clusters, while specifying a minimum cluster size of 2 (Greenbaum et al., 2011). These methods for determining MHC supertypes have been shown to identify biologically relevant variation in MHC allele functionality in both human and nonhuman primate studies (Lund et al., 2004; Schwensow et al., 2007).

### 2.4 Statistical analyses

All statistical analyses were performed in R v 4.1.0 (R Core Team, 2021).

#### 2.4.1 Aim 1: Characterize genome‐wide heterozygosity and kinship

We calculated standardized multi‐locus heterozygosity (stMLH; Coltman et al., 1999) for each individual using the “Rhh” package v1.0.1 in R (Alho et al., 2010). stMLH is defined as the proportion of genotyped loci at which an individual is heterozygous divided by the population mean heterozygosity at all genotyped loci. This metric has been used as an approximation of genome‐wide heterozygosity in numerous other studies (e.g., Bateson et al., 2016; Miller et al., 2014; Silió et al., 2013). We found no correlation between mean coverage and stMLH (*r* = .16), indicating that homozygous SNP calls are not likely to be a result of low sequencing depth and allelic dropout.

We estimated kinship between dyads using the relationship inference algorithm in the software package KING v2.2.4 (Manichaikul et al., 2010). With this method, the average estimated kinship for all pairs of individuals in the sample is set to zero, with kinship for each pair calculated by comparing to this average. Thus, kinship values can be negative when two individuals are more distantly related than the average relatedness within the population. Positive kinship values can be transformed into coefficient of relatedness (*r*) values by multiplying by a factor of 2 (Manichaikul et al., 2010).

#### 2.4.2 Aim 2: Characterize MHC heterozygosity and complementarity

To characterize the level of MHC diversity present in this population, we quantified MHC heterozygosity in seven different ways (Table 2). Given that MHC loci have undergone duplication events, and that duplicated loci are likely functionally equivalent (Klein & Figueroa, 1986), we used counts of distinct alleles and distinct supertypes, as well as quantifications of amino acid differences between alleles at each locus as measures of heterozygosity, as has been done in numerous other studies of MHC‐related mate choice (e.g., Landry et al., 2001; Reusch et al., 2001; Schwensow et al., 2007; Wegner et al., 2003). For the count‐based measures of heterozygosity (e.g. allele counts, supertype counts), we grouped class I (A and B) loci and class II (DQA and DRB) loci, due to the distinct biological functions MHC molecules of differing classes perform. When investigating amino acid‐based measures of heterozygosity, we grouped class I (A and B) loci, as they share highly similar patterns of polymorphism and originate from the same class I receptor domain (Brown et al., 1988), but we considered class II (DQA and DRB) loci separately, as DRB and DQA sequences do not share similar patterns of polymorphism, due to their origination from the β_1_ and α_1_ receptor domain, respectively.

We calculated MHC complementarity at class I and II loci separately, as the number of alleles or supertypes two individuals have in common (Table 1).

**TABLE 1:** Indices of major histocompatibility complex (MHC) heterozygosity and complementarity

**MHC heterozygosity**
1. Number of alleles (class I) 2. Number of alleles (class II)
3. Mean number of amino acid differences between alleles (MHC‐A and MHC‐B) 4. Mean number of amino acid differences between alleles (DQA) 5. Mean number of amino acid differences between alleles (DRB)
6. Number of supertypes (class I) 7. Number of supertypes (class II)
**MHC complementarity**
1. Number of shared alleles (class I) 2. Number of shared alleles (class II)
3. Number of shared supertypes (class I) 4. Number of shared supertypes (class II)

#### 2.4.3 Aim 3: Associations between diversity at different MHC classes

We statistically assessed potential associations between the diversity present at different MHC class types via linear mixed effects modeling, using the function “lmekin” in the R package “coxme” v2.2.16 (Therneau, 2020). This function is able to incorporate a kinship matrix as a random effect to account for the lack of statistical independence between data collected from related individuals. We performed two linear regressions, the first estimating the effect of the number of class I alleles on the number of class II alleles, and the second estimating the effect of the number of class I supertypes on the number of class II supertypes, including a kinship matrix as a random effect.

#### 2.4.4 Aim 4: Relationship between genome‐wide and MHC heterozygosity

We statistically assessed the concordance between genome‐wide heterozygosity and MHC‐specific measures of heterozygosity using linear mixed effects modeling and the “lmekin” function in the R package “coxme” v2.2.16 (Therneau, 2020). We performed seven linear regression models, using stMLH as the explanatory variable, one of the seven measures of an individual's MHC heterozygosity as the response variable, and a kinship matrix as a random effect. We adjusted all *p*‐values using the Benajmini and Hochberg correction for multiple hypothesis testing with the function “*p*.adjust” in the R package “stats'” v4.1.2 (Benjamini & Hochberg, 1995).

#### 2.4.5 Aim 5: Relationship between kinship and MHC complementarity

We statistically assessed the concordance between genome‐wide complementarity (i.e., kinship) to MHC complementarity by conducting robust generalized linear modeling using the R package “robustbase” v0.93.9 (Maechler et al., 2021). We ran four models, the first two using either the count of alleles in common at class I loci or class II loci as the response variable and the second two using either the count of supertypes in common at class I loci or class II loci as the response variable (Table S2). We included kinship and the total number of unique alleles or supertypes present between the dyad at the loci of interest as predictor variables. Due to the use of count data as the response variable, we used the Poisson error distribution for all models. We tested all models for collinearity of fixed effects and homoscedasticity in residual variance. Variance inflation factors for all models were less than 1.2, well below the common cutoff of 3 (Zuur et al., 2009). The residual variance structure of some models appeared to be slightly heteroscedastic, and so robust generalized linear modeling was used (Noh & Lee, 2007). We compared each full model to a reduced model excluding kinship using the robust Wald‐type test in the R package “robustbase” v0.93.9 (Ghosh et al., 2016), and corrected for multiple hypothesis testing using the Benjamini and Hochberg correction (Benjamini & Hochberg, 1995).

## 3 RESULTS

### 3.1 Aim 1: Characterize genome‐wide heterozygosity and kinship

After filtering for quality, our dataset included 103 million reads with an average of 4.4 million mapped reads per individual (Figure S2). From these sequences, STACKS assembled 59,547 loci shared between at least 18 of the 22 genotyped individuals (mean = 21.4 individuals per loci). These shared loci displayed a per individual mean coverage of 30.9 ± 13.0 SD reads per locus (min = 12.7, max = 68.7). Of these shared “RAD tag” loci, 40,983 were determined to be polymorphic, containing 77,993 SNPs in total. Pruning for SNPs in strong linkage disequilibrium removed 42,484 variants, leaving a total of 35,509 SNPs to be used in the calculation of stMLH. The percentage of SNPs identified as heterozygous in a single individual ranged from 22.7 to 27.3% (mean = 24.9 ± 1.3% SD). stMLH ranged from 0.88 to 1.09, with 1 as the average heterozygosity observed in this population of 22 study subjects.

When comparing our dataset with previously published baboon SNP data, we find that individuals in our population have similar degrees of heterozygosity to wild olive baboon individuals from populations in Aberdare, Kenya (*n* = 2, mean = 26.2 ± 1.6% SD), and Awash, Ethiopia (*n* = 27, mean = 23.9 ± 7.4% SD; Figure S3). This suggests that there has not been a substantial loss in genome‐wide heterozygosity through a genetic bottleneck or inbreeding in captivity. Furthermore, mean levels of genome‐wide heterozygosity measured in four different populations of olive baboons (2 wild: Aberdare, Kenya and Awash, Ethiopia; 2 captive: Southwest National Primate Research Center and CNRS SdP) are substantially greater than the mean levels of genetic diversity measured in wild yellow (*n* = 2), hamadryas (*n* = 2), and Kinda (*n* = 3) baboons sampled in Rogers et al. (2019) (Figure S3).

Dyadic kinship ranged from −0.21 to 0.24, corresponding to relatedness values of 0 to 0.48. Five dyads displayed relatedness equivalent to a parent–offspring or full‐sibling relationship (.25 < *r* < .5), and 11 dyads displayed kinship values equivalent to a grandparent–offspring or half‐sibling relationship (.125 < *r* < .25; Figure S4).

### 3.2 Aim 2: Characterize MHC heterozygosity and complementarity

**FIGURE 1:**
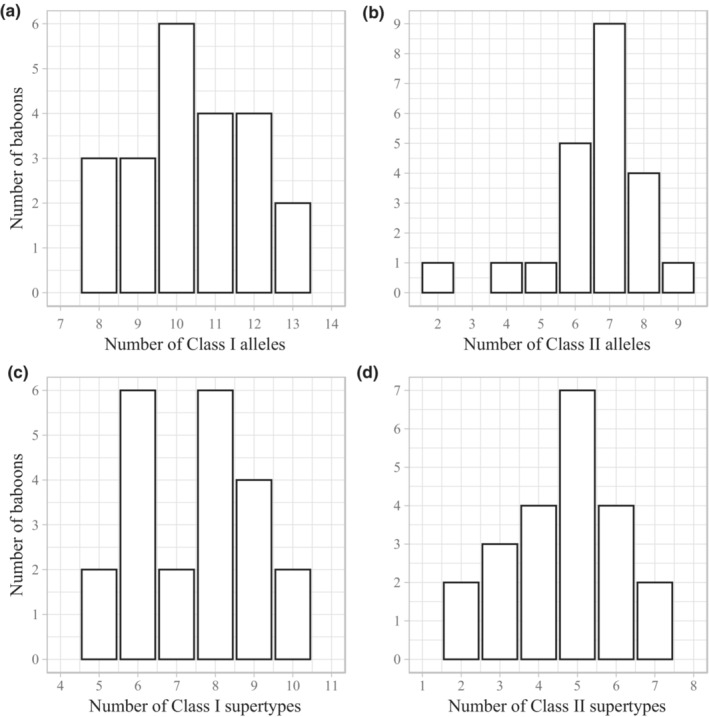
Frequency distribution of the number of (a) major histocompatibility complex (MHC) class I alleles, (b) MHC class II alleles, (c) MHC class I supertypes, and (d) MHC class II supertypes per individual in the 22 olive baboons sampled in this study.

MHC sequencing generated 8.4 million reads, with an average of 383,597 ± 47,409 SD reads per individual. Following quality filtering, sequencing depth within each individual averaged to 4031 ± 2313 SD reads per allele at A and B loci, 68,747 ± 44,932 SD reads per allele at DQA loci and 14,606 ± 7949 SD reads per allele at DRB loci. The number of both newly identified and previously described alleles identified at each locus can be found in Table 2. Each individual possessed between 8 and 13 class I (A and B) sequences (mean = 10.4 ± 1.5 SD) and between 2 and 9 class II (DQA and DRB) sequences (mean = 6.5 ± 1.5 SD; Figure 1). Dyads displayed considerable variation in their number of shared alleles. The number of shared class I alleles ranged from 0 to 11 and the number of shared class II alleles ranged from 0 to 8.

**TABLE 2:** Number of *Papio anubis* alleles sequenced per locus. Previously described alleles are those that were published on the major histocompatibility complex (MHC)‐IPD database prior to this study, and newly identified alleles are those that have not been previously published.

Loci	Previously described	Newly identified	Total
A	8	8	16
B	11	21	32
DQA	1	8	9
DRB	26	1	27
All genotyped loci	46	38	84

The number of identified PSS at each locus, the number of PSS that are orthologous to human antigen binding sites, and the number of supertypes at each locus can be found in Table S3. Additionally, aligned sequences with noted PSS and visualizations of the hierarchical clustering of alleles and resultant supertypes can be found in the Supplementary Materials (Figures S5–S10).

Each individual possessed between 10 and15 MHC supertypes (mean = 12.1 ± 1.6 SD), with 5–10 class I supertypes (mean = 7.5 ± 1.5 SD) and 2–7 class II supertypes (mean = 4.6 ± 1.4 SD; Figure 1). Dyads displayed considerable variation in their number of shared supertypes, with a range of 2–9 shared class I supertypes and 1–6 shared class II supertypes. Only two of the 22 individuals exhibited identical supertype profiles (Figure S11). Interestingly, these two individuals were not closely related (*r* = .04).

### 3.3 Aim 3: Associations between diversity at different MHC classes

An individual's genetic diversity at MHC class I loci did not exhibit a strong effect on their diversity at class II loci. This was true at both the allelic (*E*_st_ = −0.27, SE = 0.20, *z* = −1.34, *p* = .18) and supertype level (*E*_st_ = −0.25, SE = 0.20, *z* = −1.30, *p* = .19).

### 3.4 Aim 4: Relationship between genome‐wide and MHC heterozygosity

**FIGURE 2:**
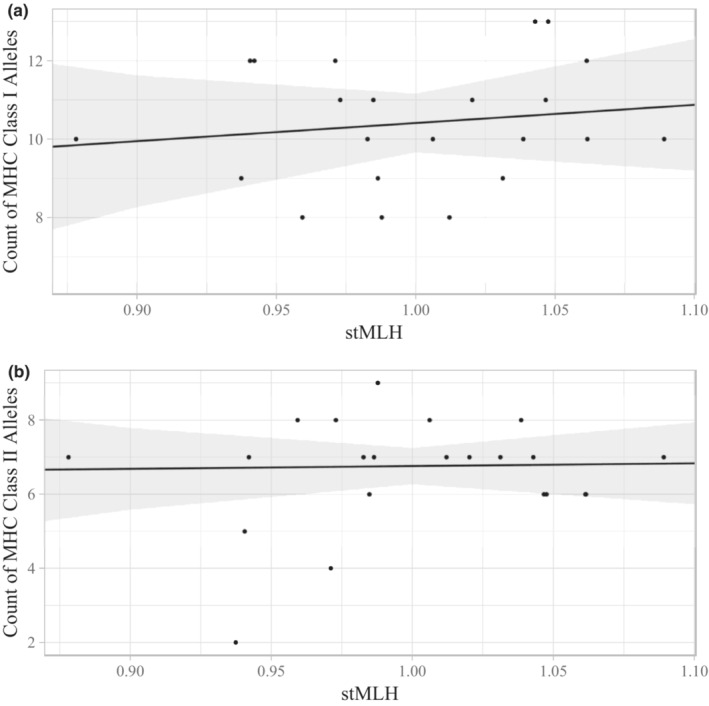
Lack of statistical relationship observed between genome‐wide heterozygosity (stMLH) and (a) major histocompatibility complex (MHC) class I allelic diversity and (b) MHC class II allelic diversity. The solid black lines represent model predictions, the shading surrounding the lines represent high and low confidence intervals, and the points represent raw data.

Genome‐wide heterozygosity (stMLH) was not a significant predictor of any of the seven measures of MHC heterozygosity in our sample (Figure 2). Full model results can be found in Table S4. This lack of relationship between genome‐wide and MHC heterozygosity was also observed when utilizing absolute heterozygosity (as opposed to stMLH), and when assessing allelic and supertype diversity at DQA and DRB loci separately.

### 3.5 Aim 5: Relationship between kinship and MHC complementarity

Kinship was a significant predictor of MHC complementarity in two of the four models. Kinship predicted the number of shared alleles between dyads at class II, but not class I loci. Likewise, kinship predicted of the number of shared supertypes between dyads at class II, but not at class I loci (Table 3).

**TABLE 3:** Robust generalized linear model results testing the effect of kinship on six different measures of major histocompatibility complex (MHC) complementarity.

Model No.	Kinship Est.	SE	*z*‐value	*p*‐value^a^	*p*‐adjust^b^
1: Alleles Class I	0.50	0.49	1.02	.31	.41
2: Alleles Class II	3.97	0.61	6.46	**<.001**	**<.001**
3: Supertypes Class I	−0.06	0.41	−0.14	.89	.89
4: Supertypes Class II	1.67	0.52	3.23	**.001**	**.002**

Significant results are in bold

^a^ *p*‐value generated from robust Wald test against null model excluding kinship.

b Adjusted for multiple hypothesis testing using the Benjamini and Hochberg correction.

## 4 DISCUSSION

In this study, we described genome‐wide and MHC diversity present in a population of olive baboons, and determined how genome‐wide measurements of heterozygosity and complementarity relate to heterozygosity and complementarity at the MHC region. Understanding the relationship between MHC genotype and genome‐wide genetic characteristics is critical for distinguishing between loci‐specific targets of female mate choice, and more general mechanisms of inbreeding avoidance and mate choice favoring genome‐wide heterozygosity. Genome‐wide heterozygosity did not display a strong effect on any of our seven measures of MHC heterozygosity, however kinship (i.e., genome‐wide genetic similarity) significantly predicted genetic similarity at class II, but not class I MHC loci. Additionally, we did not find a strong correlation between genetic diversity at class I and class II MHC loci, suggesting that the diversity present at one MHC locus or class type may not be reflective of the diversity present within an individual's MHC gene family as a whole.

The genome‐wide diversity observed in our study's sample is comparable to that observed in other captive and wild olive baboon populations, suggesting that our study's population has not experienced a substantial loss in genetic diversity due to inbreeding in captivity. We also found that the genome‐wide diversity present in our study subjects, as well as the mean diversity present other captive and wild olive baboon populations, is considerably higher than that observed in wild yellow, hamadryas, and Kinda baboons. For MHC polymorphism, we found that the baboons in our population possess similar degrees of MHC diversity to that observed in larger investigations of MHC diversity in olive baboons. A study of 154 olive baboons residing at CNRS SdP identified 1‐4 A alleles, 8‐13 B alleles (van der Wiel et al., 2018), and 4–10 DRB alleles per animal (de Groot et al., 2017), only slightly higher than the diversity measured here. The number of MHC DRB alleles observed per individual in our study's sample (mean = 5.05 ± 1.33 SD) appears similar to that observed in other baboon species (*Papio ursinus*: mean = 5.35 ± 1.6 SEM; Huchard et al., 2008), but greater than that observed in wild ring‐tailed lemurs (*Lemur catta*; mean = 2.78 ± 1.34 SD; Grogan et al., 2017), and golden snub‐nosed monkeys (*Rhinopithecus roxellana*; mean = 2.67 ± 0.78 SD; Yang et al., 2014). Ecological niche breadth and environmental heterogeneity is posited to contribute to the maintenance of genetic diversity (Habel & Schmitt, 2012; Kassen, 2002), particularly at the MHC (Qurkhuli et al., 2019), with generalist species displaying higher genetic diversity than ecological specialists. Olive baboons inhabit a particularly wide geographical range, and exhibit considerable ecological flexibility (Fischer et al., 2019), perhaps contributing to the enhanced genetic polymorphism observed in olive baboons compared to other primate taxa.

It is well documented across a wide breadth of taxa that MHC diversity does not always correlate with proxies of genome‐wide genetic diversity, including in both humans and nonhuman primates (Carrington et al., 1999; Grogan et al., 2017; Grogan et al., 2019; Sauermann et al., 2001), as well as in fish (McClelland et al., 2003; Reusch et al., 2001), birds (Promerová et al., 2011; Westerdahl et al., 2005), and other mammals (Galaverni et al., 2016). Many of these previous studies utilized a small number of neutral genetic markers (i.e., <30 microsatellite loci) to approximate genome‐wide variation, as opposed to the >35,000 genetic variants utilized here. Of the handful of studies utilizing ddRAD sequencing to investigate correlations between genome‐wide and loci‐specific diversity, the results are mixed. In the Attwater's prairie chicken (*Tympanuchus cupido attwateri*), genome‐wide heterozygosity was not associated with heterozygosity at MHC class I or class II loci (Bateson et al., 2016). However, in the three‐spined stickleback (*Gasterosteus aculeatus*), genome‐wide and MHC heterozygosity were observed to be positively correlated (Peng et al., 2021). These conflicting results may be attributed to differing amounts of sampling breadth or data analysis approaches. Peng and colleagues genotyped 1277 individuals from 26 different populations, and found an association between genome‐wide and MHC diversity when comparing population mean heterozygosity with the average number of MHC variants per population. In comparison, our study and Bateson et al. (2016) genotyped 22 and 126 individuals respectively, and found no association between genome‐wide and immune system gene diversity at the individual level. While population level degrees of diversity may covary due to shared genetic histories of gene flow or bottlenecks, it appears that genome‐wide diversity does not predict MHC diversity at the individual level. Future studies with larger sample sizes will be necessary to determine if smaller effect sizes exist, and if so, their biological relevance in mate choice processes.

In our study group, kinship was a significant predictor of genetic similarity (i.e., complementarity) at class II MHC loci. This is true when assessing complementarity as the number of shared alleles or the number of shared supertypes. This aligns with results from Savannah sparrows (*Passerculus sandwichensis*) and chacma baboons (*Papio ursinus*), both of which found that class II allele sharing increased as genome‐wide similarity increased (Freeman‐Gallant et al., 2003; Huchard et al., 2010). Interestingly, this relationship is exclusive to class II loci, the opposite pattern to that observed in the European badger (*Meles meles*; Sin et al., 2015) and Seychelles warbler (*Acrocephalus sechellensis*; Richardson et al., 2005), where relatedness covaries with class I loci exclusively. In both cases, the badger and warbler populations displayed relatively low MHC diversity in comparison to this study group, further supporting the need to understand these processes in species engaging in different mating systems and in populations exposed to variable pathogen environments.

Differences in functionality and the evolutionary forces shaping class I and class II loci could help to explain why studies of MHC‐related mate choice can often produce contrasting results depending upon which MHC genes are examined (Huchard et al., 2013). MHC class II molecules principally bind exogenous antigens derived from extracellular pathogens such as bacteria (Piertney & Oliver, 2006). Thus, MHC class II composition may play a large role in olfactory communication through its effects on the microbiome. This is supported by experimental studies in rodents, whereby class II MHC genotype influences the composition of gut microbiota (Kubinak et al., 2015), and individuals reared in germ‐free environments do not produce MHC‐associated olfactory signals (Singh et al., 1990). Although there has yet to be a study experimentally linking MHC‐associated mate choice with olfactory signaling in the olive baboon, female fertility is associated with the chemical composition of vaginal volatile compounds (Vaglio et al., 2021) and olfactory inspections of female genitalia increase during a female's fertile phase (Rigaill et al., 2013), suggesting that changes in vaginal odors are of interest to males and may influence the timing of reproductive behavior in this species. Furthermore, class II MHC genotype is associated with the chemical composition of sternal gland secretions in mandrills, further supporting the role of olfactory communication in MHC‐associated mate choice in Cercopithecine primates (Setchell et al., 2011). Conversely, class I MHC molecules bind to intracellular pathogens, such as viruses, and thus may be under different selective pressures from class II genes (Piertney & Oliver, 2006). For example, McClelland et al. (2013) found evidence of balancing and directional selection observed at class I and class II loci in sockeye salmon (*Oncorhynchus nerka*), but rarely on both loci within the same population. Furthermore, these selective pressures change from year to year, emphasizing the role of the changing pathogen environment in shaping the MHC (Westerdahl et al., 2004). Notably, all the individuals assessed in this study were born in captivity, where their microbiome composition and pathogen exposure are likely different from their wild counterparts. Thus, the selective pressures maintaining genetic diversity at class I and II loci in our study population may not reflect those present in populations evolving in more naturalistic settings.

Finally, the results presented here have numerous implications for the study of mate choice. First, they emphasize that mate choice favoring diversity at one functional locus may not always be synonymous with mate choice favoring overall genetic diversity, and vice versa. As mate choice for genome‐wide heterozygosity has been observed in numerous study systems, this suggests that (1) overall genetic diversity may be correlated to another functional locus not measured here, or (2) genome‐wide heterozygosity itself may be somehow salient and preferred by mating partners. Second, this study provides mixed support for the long‐cited hypothesis that similarity at the MHC region reflects relatedness and that MHC disassortative mating is a form of inbreeding avoidance (Brown & Eklund, 1994; Penn & Potts, 1999). Class II MHC complementarity, which may be readily communicated to others through its effects on the microbiome and olfactory signaling, is closely linked to kinship in our study sample, however class I complementarity is not. Although there are likely to be multiple mechanisms that deter inbreeding in most species, these results suggest that class II MHC‐disassortative mate choice may be one such mechanism, and emphasize the importance of controlling for relatedness if attempting to isolate the effects of MHC genotype on mate choice (Strandh et al., 2012). Third, diversity at different MHC classes does not appear to be correlated, suggesting that “allele optimizing” strategies may occur within, not between, MHC class types. Nonetheless, evidence of mate choice prioritizing immunogenetic optimality has been observed in multiple species, including the three‐spined stickleback (*Gasterosteus aculeatus*; Milinski et al., 2005), ring‐necked pheasant (*Phasianus colchicums*; Baratti et al., 2012), and golden snub‐nosed monkey (Zhang et al., 2020), among others. Further investigation of how diversity at class I and II loci interact and are coadapted for particular pathogen environments will be needed to understand the causes and consequences of diversity at these different class types.

As phenotypic data alone is often not able to explain mate choice patterns, genetics‐based mate choice has become a major interest in the field of sexual selection. As new genetic sequencing techniques develop, so does our ability to delve deeper into how genetic composition influences mate choice, offspring survival, and ultimately, fitness. This study adds clarity to how genome‐wide genetic diversity and complementarity may co‐occur with different MHC genotypes by utilizing reduced representation DNA sequencing to approximate genome‐wide genetic characteristics, and by assessing class I and class II MHC loci separately. Furthermore, this study highlights how different selective pressures may maintain diversity at class I and II loci, perhaps due to their differing roles in the immune system and/or olfactory communication. Local parasite adaptation and mating system will both likely play large roles in shaping the MHC, and thus these patterns may be species and/or population specific. It will be important moving forward to understand how genome‐wide diversity relates to genetic diversity at functional loci of interest within each study system to appreciate how mate choice may act to maximize offspring success in these different contexts.

## AUTHOR CONTRIBUTIONS

**Rachel M. Petersen:** Conceptualization (equal); formal analysis (lead); funding acquisition (equal); writing – original draft (lead); writing – review and editing (lead). **Christina M. Bergey:** Methodology (equal); supervision (equal); writing – review and editing (equal). **Christian Roos:** Methodology (equal); supervision (equal); writing – review and editing (equal). **James P. Higham:** Conceptualization (equal); funding acquisition (equal); supervision (equal); writing – review and editing (equal).

## CONFLICT OF INTEREST

The authors have no conflicts of interest to disclose.

## Supporting information


Appendix S1

## Data Availability

The genetic sequences generated in this study are openly available in the Sequence Read Archive, project number PRJNA875430 (ddRAD sequences) and in GenBank, accession numbers OP375715–OP375798 (MHC sequences); Code is available on Dryad (https://doi.org/10.5061/dryad.j3tx95xj8) and R.M.P.'s personal GitHub page: https://github.com/rachpetersen/globalvsMHC.git.
