# Supplementary material for: Relationship between genome‐wide and MHC class I and II genetic diversity and complementarity in a nonhuman primate
Source: Ecol Evol. 2022 Oct 13;12(10):e9346. doi: 10.1002/ece3.9346 (PMC9596323; doi:10.1002/ece3.9346)
Supplement: Supplementary file 1 — Appendix S1 [file ECE3-12-e9346-s001.docx]

**Supplementary Materials for: “Relationship between genome-wide and MHC class I and II genetic diversity and complementarity”**

**Rachel M. Petersen, Christina M. Bergey, Christian Roos, and James P. Higham**

**Table of Contents:**

| Text S1 | Page 2-3 |
| --- | --- |
| Table S1 | Page 3 |
| Table S2- S5 | Page 4 |
| Table S6 | Page 5 |
| Figure S1 | Page 6 |
| Figure S2 | Page 7 |
| Figure S3 | Page 8 |
| Figure S4 | Page 9 |
| Figure S5 | Page 10 |
| Figure S6 | Page 11 |
| Figure S7- S8 | Page 12 |
| Figure S9 | Page 13 |
| Figure S10- S11 | Page 14 |
|  |  |

Text S1: Comprehensive laboratory methods

*Genome-wide SNP genotyping by sequencing*

We conducted double digest restriction-site associated DNA sequencing (ddRADseq) library preparation following Peterson et al. (2012). We digested 1µg of DNA using 10 units of two restriction enzymes (SphI and MluCI), and excluded fragments outside of a 185 ± 19 bp target window using the automated Blue Pippin System and 2% Agarose Gel Cassettes. We chose these enzymes because their cut site frequency was predicted to generate approximately 40,000 fragments at the desired fragment length, consisting of approximately 0.3% of the diploid genome (Peterson et al., 2012). Due to the small number of individuals being sequenced, we ligated non-barcoded adapters designed for the Illumina platform customized for enzyme cut sites and cleaned products using AMPure XP beads at a ratio of 0.75. We individually indexed samples using NEBNext Multiplex Oligos for Illumina sequencing with the following cycling conditions: initial denaturation at 95°C for 2 minutes, 8 cycles of denaturation at 95°C for 30 seconds, annealing at 60°C for 30 seconds, extension at 72°C for 75 seconds, and a final extension at 72°C for 10 minutes. We then combined samples into 5 pools and conducted a second round of size selection on the Blue Pippin system, selecting 340 ± 63 bp fragments to account for the length of the adapters. We determined the molarity of each pool using qPCR, and diluted the pools into one 2nM library, which was sequenced on one lane of the Illumina HiSeq 2500 in 150 bp paired end reads.

*MHC sequencing*

We amplified and sequenced functionally significant regions of four MHC receptor types, representing two classes of MHC molecules: MHC A and B (class I), and MHC DQ and DR (class II). Within class I loci, we sequenced a 195 bp segment within the α_1_ domain involved in antigen binding. Within class II loci, we sequenced a 188 bp segment within the α_1_ domain of DQ receptors (i.e. DQA) and a 252 bp segment within the β_1_ domain of DR receptors (i.e. DRB: Figure 1). These segments comprise portions of the antigen binding cleft, and hence include amino acids that are functionally important for the recognition and binding of intra and extracellular pathogens.

We used the MilliporeSigma FastStart High Fidelity PCR System, 50ng of template DNA, and primers (Metabion) described in Table S2. We forwent barcoded adapters and used identical adapters (forward: 5’-ACACTCTTTCCCTACACGACGCTCTTCCGATCT-3’; reverse: 5’-GTGACTGGAGTTCAGACGTGTGCTCTTCCGATCT-3’) for all individuals and added individual specific indexing during PCR amplification. Cycling conditions were as follows: initial for 2 minutes at 95°C, 30 cycles of denaturation for 30 seconds at 95°C, annealing for 30 seconds at 52°C for DQA, 54°C for DRB and 60°C for AB loci, extension for 1 minute at 72°C, and a final extension for 5 minutes at 72°C. We decreased the volume of the PCR products in a vacuum centrifuge, and placed whole product with 1ul of loading dye in 1.5% agarose gel at 130V for 15 minutes. We excised the appropriate bands and extracted the PCR product from the gel using the Monarch DNA gel extraction kit. We estimated the concentration of the PCR products on Qubit, estimated molarity, and performed an indexing PCR using Hot Start Pfu DNA Polymerase, 2ul of 2.9nM loci specific PCR product, 22 primer pairs (one unique pair for each individual), and the following cycling conditions: initial denaturation at 98°C for 30 seconds, 8 cycles of denaturation at 98°C for 10 seconds, annealing at 62°C for 20 seconds, extension at 72°C for 20 seconds, and a final extension at 72°C for 10 minutes. We cleaned the indexed products using AMPure XP beads at a ratio of 0.7, reamplified if necessary to reach a 3nM concentration for each locus for each individual, and pooled all individuals to make a final 3nM library for sequencing on one lane of the Illumina MiSeq in 200bp paired end reads.

**Table S1.** Origination of study individuals’ parents and grandparents. Unknown information is designation by a question mark. Individuals from Kenya are those that were born within an unknown wild population in Kenya.

| **Study Individual** | **Mother** | **Maternal origin** | **Maternal grandmother** | **Maternal grandfather** | **Maternal grandparent origin** | **Father** | **Paternal origin** | **Paternal grandmother** | **Paternal grandfather** | **Paternal grandparent origin** |
| --- | --- | --- | --- | --- | --- | --- | --- | --- | --- | --- |
| Ursuline | June | St Vrain Zoo | ? | ? | ? | Ivar | St Vrain Zoo | ? | ? | ? |
| Virginie | Helda | St Vrain Zoo | ? | ? | ? | ? | ? | ? | ? | ? |
| Auguste | Jesie | St Vrain Zoo | ? | ? | ? | Ivar | St Vrain Zoo | ? | ? | ? |
| Alf | Fara | St Vrain Zoo | ? | ? | ? | Gaspard | St Vrain Zoo | ? | ? | ? |
| Epine | Vanessa | CNRS SdP | Grace | Gaspard | St Vrain Zoo | ? | ? | ? | ? | ? |
| Bibi | Hue | Kenya | ? | ? | ? | Kiwi | St Vrain Zoo | ? | ? | ? |
| Barbie | Naomie | St Vrain Zoo | ? | ? | ? | Kiwi | St Vrain Zoo | ? | ? | ? |
| Salade | Fara | St Vrain Zoo | ? | ? | ? | Gaspard | St Vrain Zoo | ? | ? | ? |
| Sottise | Hotte | Kenya | ? | ? | ? | Alex | Kenya | ? | ? | ? |
| Rodolphe | Heli | St Vrain Zoo | ? | ? | ? | Paul | SdP | Babe | ? | St Vrain Zoo |
| Actu | Grace | St Vrain Zoo | ? | ? | ? | Gaspard | St Vrain Zoo | ? | ? | St Vrain Zoo |
| Osiris | Vroni | St Vrain Zoo | ? | ? | ? | Gaspard | St Vrain Zoo | ? | ? | ? |
| Viva | Ora | St Vrain Zoo | ? | ? | ? | Olav | CNRS SdP | Vroni | ? | St Vrain Zoo |
| Vuei | Olive | CNRS SdP | Effi | Melchior | St Vrain Zoo | Momo | CNRS SdP | Ida | Jojo | ? |
| Vertige | Pomponette | CNRS SdP | Fara | Gaspard | St Vrain Zoo | Momo | CNRS SdP | Ida | Jojo | ? |
| Solene | Ibis | St Vrain Zoo | ? | ? | ? | Tarzan | Kenya | ? | ? | ? |
| Odila | ? | ? | ? | ? | ? | ? | ? | ? | ? | ? |
| Rubis | Flura | St Vrain Zoo | ? | ? | ? | ? | ? | ? | ? | ? |
| Suzon | Katy | CNRS SdP | Julie | Jojo | Paris Zoo | Idefix | Marseille Zoo |  |  | ? |
| Momo | Ida | CNRS SdP | ? | ? | ? | Jojo | Paris Zoo | ? | ? | ? |
| Raimu | Jesie | St Vrain Zoo | ? | ? | ? | ? | ? | ? | ? | ? |
| Marius | GR3 | Grenoble Zoo | ? | ? | ? | Sylvestre | Paris Zoo | ? | ? | ? |

**Table S2.** Primer sequences used to amplify MHC A, B, DQA, and DRB loci

| **Loci** | **Primer sequences (forward and reverse)** | **fragment**  **length** | **Reference (species)** |
| --- | --- | --- | --- |
| A & B | 5′TACGGTAGCAGAGACTTGGTCTGGGCTA  CGTGGACGACAC | 195 bp | Morgan et al. 2018 (*P. anubis*) |
|  | 5′ACACTGACGACATGGTTCTACAGCCTCG  CTCTGGTTGTAGTAG |  |  |
| DQA | 5′GTGCTGCAGGTGTAAACTTGTACCAG | 188bp | Mwenda et al. 1997 (*P. anubis*);  Alberts 1999 (*P. cynocephalus* & *P. papio*) |
|  | 5′CACGGATCCGGTAGCAGCGGTAGAGTTG |  |  |
| DRB | 5′GCCTCGAGTGTCCCCCCAGCACGTTTC | 252bp | Knapp et al. 1997 (*M. mulatta*) ; Huchard et al. 2006 (*P. ursinus*) |
|  | 5′GCAAGCTTTCACCTCGCCGCTG |  |  |

**Table S3.** Robust generalized linear models testing the effect of kinship on six different measures of MHC complementarity

| **Model No.** | **Response** | **Predictor** | **Covariate** | **Family** |
| --- | --- | --- | --- | --- |
| 1 | Count of class I alleles in common | Kinship | Count of unique class I alleles | Poisson |
| 2 | Count of class II alleles in common | Kinship | Count of unique class II alleles | Poisson |
| 3 | Count of class I supertypes in common | Kinship | Count of unique class I supertypes | Poisson |
| 4 | Count of class II supertypes in common | Kinship | Count of unique class II supertypes | Poisson |

**Table S4.** Summary statistics of supertype analyses. Number of positively selected sites (PSS) identified at each locus, the number of *Papio anubis* PSS that are orthologous to human HLA antigen binding sites (ABS), and the number of resultant supertypes determined using hierarchical clustering and dynamic branch cutting.

| **Loci** | **PSS** | **HLA ABS orthologs** | **Supertypes** |
| --- | --- | --- | --- |
| A/B | 16 | 8 | 12 |
| DQA | 4 | 3 | 3 |
| DRB | 18 | 13 | 6 |

**Table S5.** Linear mixed model estimates of the effect of MHC class I diversity on MHC class II diversity.

| **Model** | **Predictor variable** | **Response variable** | **Estimate** | **Std. Error** | **z-value** | **p-value** |
| --- | --- | --- | --- | --- | --- | --- |
| 1 | Number of class I alleles | Number of class II alleles | -0.27 | 0.20 | -1.34 | 0.18 |
| 2 | Number of class I supertypes | Number of class II supertypes | -0.25 | 0.20 | -1.30 | 0.19 |

**Table S6.** Linear mixed model estimates of the effect of global heterozygosity (stMLH) on seven different measures of MHC heterozygosity.

| **Model** | **Predictor variable** | **Response variable** | **Estimate** | **Std. Error** | | **z-value** | **p-value** | **p-adjust*** |
| --- | --- | --- | --- | --- | --- | --- | --- | --- |
|  |  | *Count of alleles at…* |  |  |  | |  |  |
| 1 | Global heterozygosity | Class I loci | 5.92 | 6.50 | 0.91 | | 0.36 | 0.63 |
| 2 | Global heterozygosity | Class II loci | 6.66 | 7.06 | 0.94 | | 0.35 | 0.63 |
|  |  | *Mean number of amino acid differences at…* |  |  |  | |  |  |
| 3 | Global heterozygosity | AB alleles | -0.15 | 3.85 | -0.04 | | 0.97 | 0.97 |
| 4 | Global heterozygosity | DQA alleles | 40.35 | 30.69 | 1.31 | | 0.19 | 0.63 |
| 5 | Global heterozygosity | DRB alleles | 21.42 | 16.34 | 1.31 | | 0.19 | 0.63 |
|  |  | *Count of supertypes at…* |  |  |  | |  |  |
| 6 | Global heterozygosity | Class I loci | 4.52 | 6.56 | 0.69 | | 0.49 | 0.69 |
| 7 | Global heterozygosity | Class II loci | 2.64 | 6.30 | 0.42 | | 0.68 | 0.79 |

* adjusted for multiple hypothesis testing using the Benjamini and Hochberg correction

**Figure S1.** Mean intra-individual drops in sequence copy number when comparing the sequence with the fewest reads falling above the 1,000 read threshold to the sequence with the most reads falling below the 1,000 read threshold. For example, when looking at the number of MHC-A sequences within each individual, the sequence with the fewest reads falling above the threshold has on average 760 more read copies than the sequence with the next most read copies falling below the threshold. This natural drop in sequence copy numbers is most pronounced in DQA sequences, where individuals had an average drop of 62,665 read copies when comparing the sequence falling just above to the sequence falling just below the 1,000 read cutoff.

**Figure S2.** Number of ddRADseq reads mapping to the NCBI Panu v. 3.0 genome, with a mean of 4.4 ± 2 million mapped reads per individual (range= 10.3 -1.97 million).

**Figure S3.** Variation in the percentage of SNPs identified as heterozygous in *Papio spp.* individuals originating from 9 different populations. SNP heterozygosity was measured at 27,303 ± 479 (sd.) loci per individual, which overlapped between the SNP dataset generated in this study and two previously published datasets (Bergey, 2015; Rogers et al., 2019). Wild populations are indicated by their geographic location and captive populations are designated by the acronym of the research institution (CNRS: le Centre National de la Recherche Scientifique; SNPRC: Southwest National Primate Research Center). Individuals in our study population (CNRS) have comparable degrees of heterozygosity to that observed in other wild and captive olive baboons, and greater heterozygosity than that found in wild and captive populations of other baboon species.

**Figure S4.** Relatedness matrix for 22 olive baboon study subjects calculated from 77,993 SNPs generated by double digest restriction site associated DNA (ddRAD) sequencing.


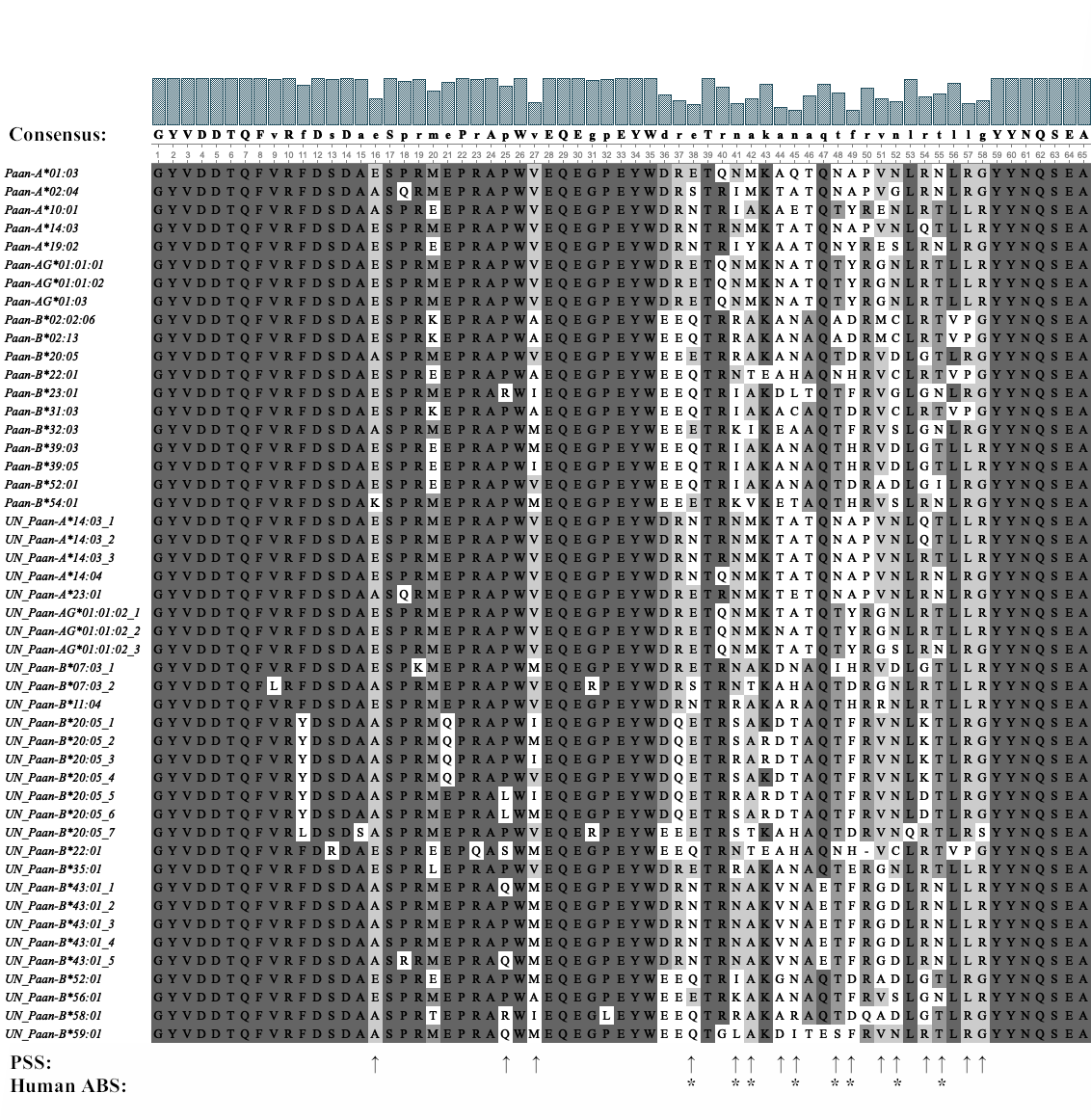


**Figure S5.** MHC A and B aligned amino acid sequences. The grayscale of the sequences reflects percent identity with darker colors signifying a higher consensus between sequences. **↑** signifies positively selected sites (PSS) identified in this study (positions 16, 25, 27, 38, 41, 42, 44, 45, 48, 49, 51, 52, 54, 55, 57, 58). * indicates which positively selected sites are in agreement with antigen binding sites (ABS) identified in the human HLA-A molecule (Bjorkman et al. 1987). Previously known alleles are identified using standard MHC nomenclature (Ellis et al. 2006) and previously unidentified alleles are designated by “UN_” followed by the standard nomenclature for the most similar known allele.

**Figure S6.** Twelve MHC A and B supertypes identified using the physiochemical properties of sixteen positively selected sites and agglomerative hierarchical clustering using Euclidean distance and average linking method.


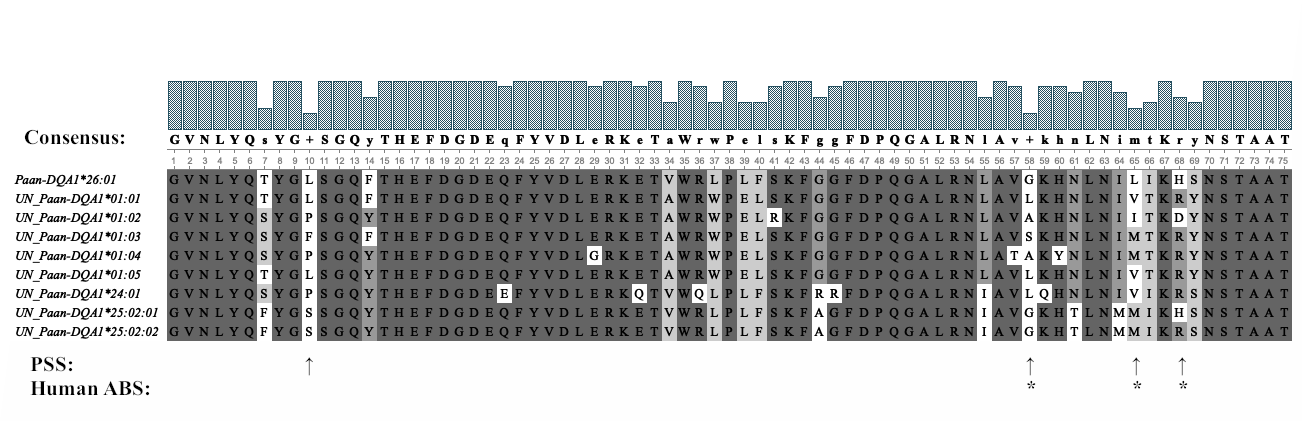


**Figure S7.** MHC DQA aligned amino acid sequences. The grayscale of the sequences reflects percent identity with darker colors signifying a higher consensus between sequences. **↑** signifies positively selected sites (PSS) identified in this study (positions 10, 58, 65, 68). * indicates which positively selected sites are in agreement with antigen binding sites (ABS) identified in the human HLA-DQ molecule (Brown et al. 1988, 1993).

**Figure S8.** Three MHC-DQA supertypes identified using the physiochemical properties of four positively selected sites and agglomerative hierarchical clustering using Euclidean distance and average linking method.


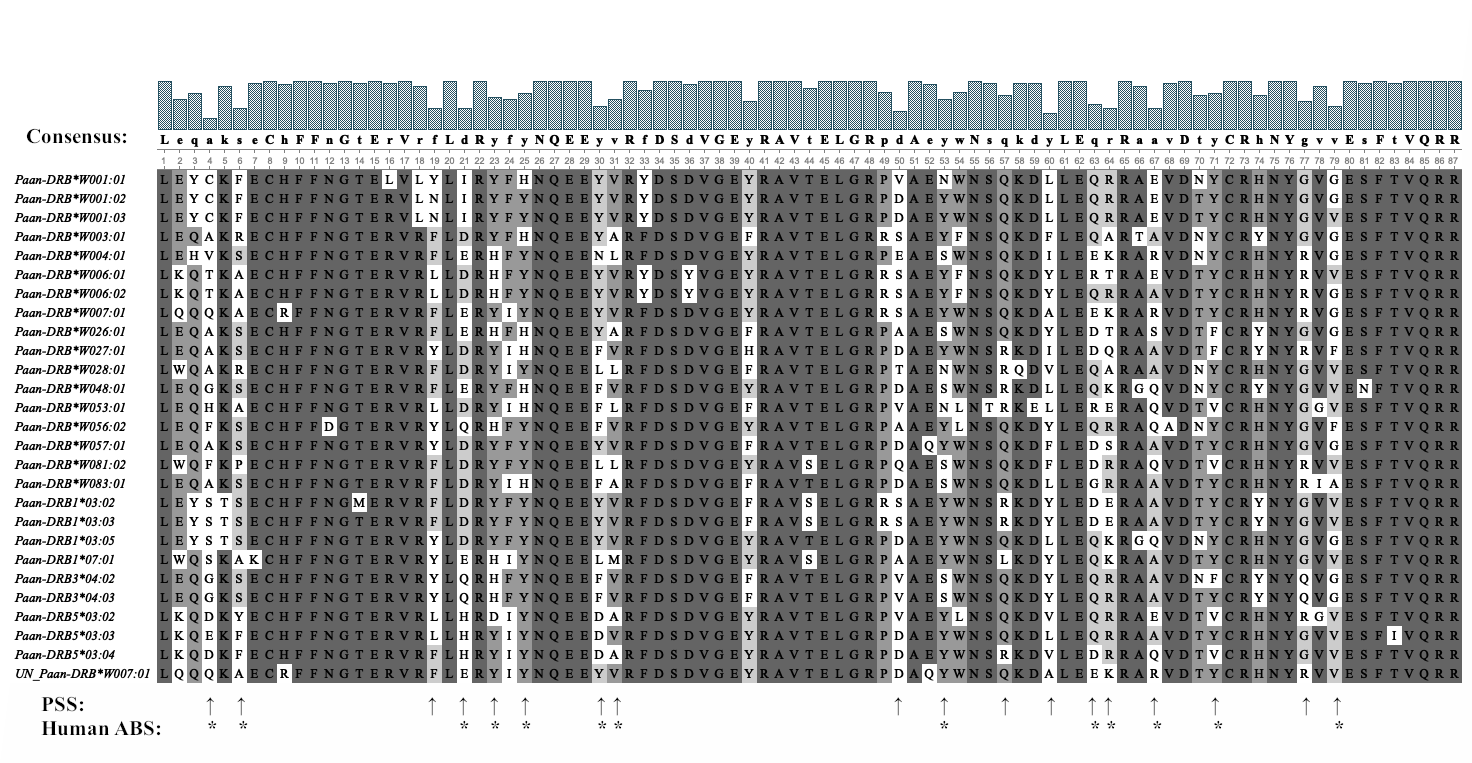


**Figure S9.** MHC DRB aligned amino acid sequences. The grayscale of the sequences reflects percent identity with darker colors signifying a higher consensus between sequences. **↑** signifies positively selected sites (PSS) identified in this study (positions 4, 6, 19, 21, 23, 25, 30, 31, 50, 53, 57, 60, 63, 64, 67, 71, 77, 79). * indicates which positively selected sites are in agreement with antigen binding sites (ABS) identified in the human HLA-DR molecule (Brown et al. 1988, 1993).

**Figure S10.** Six MHC DRB supertypes identified using the physiochemical properties of eighteen positively selected sites and agglomerative hierarchical clustering using Euclidean distance and average linking method.

**Figure S11.** Variation in supertype profiles of 22 olive baboon study subjects.

Bergey, C. (2015). Population genomics of a baboon hybrid zone. *New York: New York University*.

Peterson, B. K., Weber, J. N., Kay, E. H., Fisher, H. S., & Hoekstra, H. E. (2012). Double Digest RADseq: an inexpensive method for de novo SNP discovery and genotyping in model and non-model species. *PloS One, 7*(5), e37135. doi:10.1371/journal.pone.0037135

Rogers, J., Raveendran, M., Harris, R. A., Mailund, T., Leppälä, K., Athanasiadis, G., . . . Walker, J. A. (2019). The comparative genomics and complex population history of Papio baboons. *Science Advances, 5*(1), eaau6947.
